# What do the data say about chemotherapy-induced peripheral neuropathy assessment and management?

**DOI:** 10.1016/j.apjon.2022.100170

**Published:** 2022-11-24

**Authors:** Grace Kanzawa-Lee, Robert Knoerl

**Affiliations:** University of Michigan School of Nursing, Ann Arbor, MI, USA

## Why is chemotherapy-induced peripheral neuropathy a problem?

Chemotherapy-induced peripheral neuropathy (CIPN) affects nearly 7 in 10 patients receiving neurotoxic chemotherapy treatment (eg., taxanes, platinums, vinca alkaloids). The symptoms of CIPN include numbness (reduced sensation and proprioception), tingling, pain and/or weakness in the bilateral upper and lower extremities and may negatively affect physical function during and in the years following chemotherapy completion. Due to the lack of effective CIPN treatments, clinicians and patients are faced with the difficult decision of whether to stop chemotherapy early to decrease CIPN progression and preserve patients’ physical function but risk suboptimal tumor-treating chemotherapy dosages.

Despite the noted negative impact of CIPN on patient-reported outcomes, many unanswered questions and practice gaps surround CIPN assessment and management. There are no gold standard measures, no preventative treatments and only one recommended pharmacological treatment. However, new evidence is emerging regularly. The purpose of this editorial is to provide clarity surrounding the current evidence on CIPN assessment and management in clinical practice.

## What do the data tell us about CIPN assessment?

A primary barrier to establishing appropriate CIPN management has been the persistent practice of relying solely on clinician-based grading scales (eg., National Cancer Institute Common Terminology Criteria for Adverse Events [CTCAE]) to assess CIPN despite clear evidence of their psychometric limitations and poor sensitivity and reliability.^1^ Thus, an immediate goal for advancing clinical practice is to transition from using just clinician-graded scales to systematically implementing evidence-based CIPN measures in the clinical setting.

Recent evidence suggests that a combination of clinician assessment and patient-reported outcome (PRO) measures are necessary to adequately assess CIPN.^1^ The clinician may periodically assess for changes in peripheral extremity strength, deep tendon reflexes, and vibration sensibility. The 5-item Total Neuropathy Score – Clinical Version (TNSc©)^2^ is one recommended clinician-based assessment to measure sensory and motor symptoms, strength, deep tendon reflexes, and vibration sensibility.^1^ However, clinician-based assessments may be difficult to systematically implement in the clinical setting due to its time and clinician training requirements for proper use.^3^

While there is no consensus regarding the best CIPN assessment measures,^1^ shorter PRO scales may be preferred by patients and clinicians to longer time-consuming PRO scales and unwieldy clinician-based assessments. For example, the PRO-CTCAE offers two items to assess numbness and tingling severity and interference, which may help to standardize CIPN screening in the clinical setting.^4^ Preliminary evidence supports the reliability and validity of the PRO-CTCAE numbness and tingling severity and interference items for CIPN screening.^5^

Clinicians should assess for neuropathy signs and symptoms pre-, peri-, and post-chemotherapy treatment. Assessment should continue for at least six months following neurotoxic chemotherapy treatment completion as patients may develop chronic CIPN even after completing the chemotherapy regimen.

Despite the lack of a gold-standard CIPN measure, clinicians can utilize the evidence-based measures to inform their assessment. For example, nurses can use simple questioning to assess for sensory and motor impairments:

“Do you have any numbness, tingling, ‘pins and needles’ sensations and/or burning, freezing, electric shock-like pain in your hands or feet?”

“Do you feel like your arms or legs have ‘fallen asleep’?”

“Do you have any difficulty with completing tasks, such as buttoning a shirt, using a fork, knife, or pen, typing, opening a jar, or walking?”

Giving examples of sensations can improve assessment, particularly of patients who may otherwise have difficulty describing their symptoms. Finally, nurses can observe patients’ gait and assess hand grip, wrist extension, ankle dorsiflexion strength. By documenting these assessment findings, clinicians can better monitor CIPN trends over time.

## What do the data tell us about CIPN management?

Duloxetine 60 ​mg/day (starting with 30 ​mg/day then progressing to 30 ​mg twice daily) is the only recommended pharmacological treatment for the management of chronic CIPN pain.^6^ However, data suggest that duloxetine is not routinely administered in practice due to clinicians being more familiar with gabapentin, insurance barriers, and/or concern for drug–drug interaction.^1^ There are no pharmacological recommendations for the management of non-painful sensory CIPN (eg., numbness or tingling) or the prevention of CIPN.^6^

Many non-pharmacological agents and interventions have been tested, and only physical exercise (particularly endurance training) has demonstrated modest benefit in preventing CIPN based on moderate evidence.^7^^,^^8^ Sensorimotor (balance) training may be explored for the prevention and management of CIPN based on its safety and moderate-level evidence of its efficacy in improving CIPN symptoms and balance.^8^ Regular screening for patient physical activity can be operationalized with tools such as the Physical Activity Vital Sign.^9^ Further, assessing for functional and balance disturbances can inform whether an individual requires referral to a physical therapist or clinical exercise physiologist. Most individuals can safely perform exercise at home and may only require encouragement and prescription to move toward meeting or maintaining the physical activity guidelines; an example exercise prescription form can be found online.^10^ Finally, clinicians can advise patients about simple balance training exercises that they can do at home, such as tandem walking, standing on one foot, and walking on soft surfaces.

Cryotherapy (cooling of the hands and feet) has not demonstrated sufficient efficacy in preventing CIPN; rather, studies have primarily shown patient discomfort from and poor tolerance to cryotherapy. Despite the lack of evidence, cryotherapy is increasingly observed in the infusion setting; thus, clinicians must be alert and ensure that cryotherapy is only used for patients receiving paclitaxel or docetaxel, as use of cryotherapy during platinum-based chemotherapy (eg., oxaliplatin) could cause further harm.^8^ Several other non-pharmacological interventions not supported for the prevention or treatment of CIPN include neurofeedback, electrical stimulation, massage therapy.^8^ Non-pharmacological interventions that are currently under study for the prevention or treatment of CIPN, but require further evidence to demonstrate efficacy, include yoga, acupuncture, and transcutaneous electrical nerve stimulation.

**Fig. 1:**
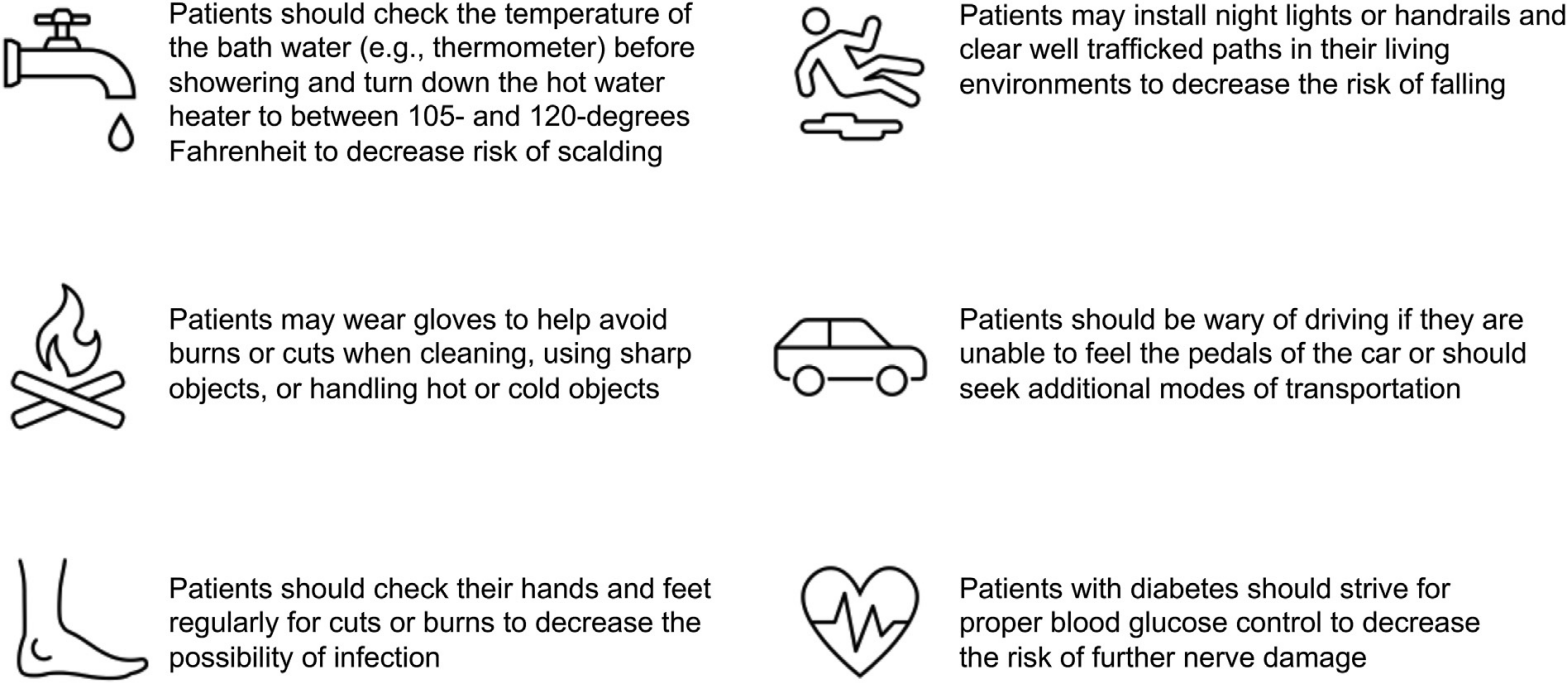
Safety tips for patients with CIPN. Reprinted with permission of Ref 11.

In addition to providing CIPN treatments, clinicians can help inform patients about the expected CIPN symptom trajectory and safety tips. Safety tips may include good foot care, fall-proofing the environment, and using gloves to handle sharp and/or cold objects (Fig. 1).^11^

## Conclusions

CIPN continues to be a key dose-limiting side effect of neurotoxic chemotherapy regimens. Consistent and intentional assessment of CIPN is necessary before, during, and after neurotoxic chemotherapy completion. The first step clinicians can take to improve CIPN management is to screen patients by interview or brief survey for numbness, tingling, burning/freezing/zapping pain in the bilateral hands and feet and difficulty with daily tasks that involve use and strength of the hands and feet. Proactive screening can help identify developing CIPN among patients who may have difficulty describing or are hesitant to report their symptoms.

The appropriate management option for chronic CIPN pain is duloxetine. Physical exercise may be useful for both prevention and treatment of CIPN and its associated functional deficits, but is not currently included in CIPN practice guidelines.^6^ Simple home exercise prescription and recommendations are safe for many but not all patients; referral to a physical therapist or clinical exercise physiologist may be most appropriate for individuals with significantly compromised balance and physical function. Multiple resources (see reference list) are available to aid clinicians in assessing for, managing, and educating patients about CIPN.

## Funding

Nil.

## Declaration of competing interest

**GKL** declares no competing interests. **RK** has received personal fees (consulting) from Strategy Inc, Spark Healthcare, Fors Marsh Group, Osmol Therapeutics, Inc., and the Comprehensive and Integrative Medicine Institute; and serves on the scientific advisory board of Wellium.
